# Reply to Letter to the Editor on ‘Smoking cessation in pregnancy: An update for maternity care practitioners’

**DOI:** 10.18332/tid/118164

**Published:** 2020-03-26

**Authors:** Paraskevi A. Katsaounou, Sophia Papadakis

**Affiliations:** 1 National and Kapodistrian University of Athens, Athens, Greece; 2 Department of Pulmonary Medicine and Respiratory Failure, First ICU, Evaggelismos Hospital, Athens, Greece; 3 Division of Cardiology, Faculty of Medicine, University of Ottawa, Ottawa, Canada; 4 Division of Prevention and Rehabilitation, University of Ottawa Heart Institute, Ottawa, Canada

**Keywords:** smoking, pregnant women, smoking cessation, nicotine replacement therapy

We would like to express our delight that our publication has gained the attention of other members of the clinical and scientific community. In his letter to the editor, A. Braillon highlighted the need for the clinical community to more effectively engage pregnant women who smoke, something that we could not agree with more.

Braillon notes that treatment models such as the 5 As are not used in his own clinical practice and he raises concerns about the model. While we appreciate the message of ‘treat without delay’ that is being communicated, we would like to clarify our perspective regarding the 5 As model to readers. The 5 As model offers clinicians distinct actions that can be used to address tobacco use in clinical settings, beginning with the systematic identification of women who smoke, offering advise on quitting, information on the risks to the fetus, and linking women to evidence-based treatment to support quitting as early as possible in their pregnancy. Experienced clinicians, who feel very comfortable treating tobacco use in patients, may prefer to use less structured counselling approaches. However, this should not detract from the value of models such as the 5 As for guiding smoking cessation treatment among clinicians who may be less experienced in working with smokers and/or institutions, and who are seeking to create protocols to support tobacco treatment delivery among pregnant women.

Braillon also raises concerns regarding the possibility that overemphasizing the harmful effects of nicotine on the fetus could unintentionally serve as a barrier to effective smoking cessation in pregnancy. We agree that one should not deprive pharmaceutical treatment to pregnant women who are addicted to nicotine and that the evidence supports the risk benefit of NRT use, which profoundly outweighs continued tobacco use. There has been significant progress in terms of the conservative guidance regarding the use of NRTs among pregnant women in the past and we expect we will see new research and guidance in this area in the next 2–3 years. The current recommendation is that NRT should be used in order to achieve complete abstinence from smoking among women who are unable to quit. The poor compliance with NRT treatment that has been documented among pregnant women suggests that attention needs to be paid to increasing clinicians’ and women’s confidence in terms of NRTs role in supporting cessation among pregnant women. Given the benefits in the first trimester of pregnancy, treatment should be initiated without delay.

We note that since our review was accepted for publication, an updated NRT product monograph was published by the Electronic Medicines Compendium (EMC)^1^ in the United Kingdom, which states that ‘NRTs should be used if the mother cannot (or is considered unlikely to) quit without pharmacological support, as the risk to the fetus is lower than that expected with smoking tobacco’. The updates to the EMC product monograph are a step in the right direction for clearer guidance to the clinical community and women on the use of NRT in pregnancy.
